# Analysis of Volatile Markers and Their Biotransformation in Raw Chicken during *Staphylococcus aureus* Early Contamination

**DOI:** 10.3390/foods12142782

**Published:** 2023-07-21

**Authors:** Yin Wang, Xian Wang, Yuanyuan Huang, Tianli Yue, Wei Cao

**Affiliations:** Department of Food Science, College of Food Science and Technology, Northwest University, Xi’an 710069, China; 17803392183@163.com (X.W.); 15093665339@163.com (Y.H.); caowei@nwu.edu.cn (W.C.)

**Keywords:** VOCs, *Staphylococcus aureus*, HS-GC-IMS, HS-SPME-GC-MS, chicken

## Abstract

To address the potential risks to food safety, headspace solid-phase microextraction coupled with gas chromatography-mass spectrometry (HS-SPME-GC-MS) and headspace gas chromatography–ion mobility spectrometry (HS-GC-IMS) were used to analyze the volatile organic compounds (VOCs) generated from chilled chicken contaminated with *Staphylococcus aureus* during early storage. Together with the KEGG database, we analyzed differential metabolites and their possible biotransformation pathways. Orthogonal partial least squares discriminant analysis (OPLS-DA) was applied to characterize VOCs and identify biomarkers associated with the early stage of chicken meat contamination with *S. aureus*. The results showed 2,6,10,15-tetramethylheptadecane, ethyl acetate, hexanal, 2-methylbutanal, butan-2-one, 3-hydroxy-2-butanone, 3-methylbutanal, and cyclohexanone as characteristic biomarkers, and 1-octen-3-ol, tetradecane, 2-hexanol, 3-methyl-1-butanol, and ethyl 2-methylpropanoate as potential characteristic biomarkers. This provides a theoretical basis for the study of biomarkers of *Staphylococcus aureus* in poultry meat.

## 1. Introduction

Chicken is the second most abundant source of edible meat protein, has a high edible and nutritional value, and is widely popular among consumers [1]. Global chilled poultry production was projected to reach 121 million tons in 2021, representing an increase in both demand and trade as a proportion of total meat consumption. China, the world’s second largest chicken producer, produced 15.42 million tons of chilled chicken in 2021. The shelf life of fresh poultry is limited as it is susceptible to spoilage due to pathogen contamination even in refrigeration. With its high levels of protein, fat, vitamins, inorganic elements, and moisture, it provides an ideal medium for microbial survival and growth during processing [2].

*Staphylococcus aureus* (*S. aureus*) is the second most common source of infection after *E. coli* in foods, primarily found in meat, milk, egg products, and baked goods [3,4,5]. The percentage of *S. aureus* isolated from chicken hoof dermatitis in broiler chicken was 68% [6]. Presence of *S. aureus* in these contaminated chicken meats and products may cause the production of heat-stable enterotoxins, increasing the likelihood of staphylococcal food poisoning in humans. Detection of *S. aureus* in chicken meat found 66.7% (80/120) and 34% infection rates, respectively [7,8]. A dramatic economic burden has resulted from the contamination of poultry meat with *S. aureus*, resulting in early detection of *S. aureus* in poultry meat becoming a matter of priority.

Volatile organic compound (VOC) analysis is an accepted method for monitoring food quality because the microbial metabolism produces many VOCs during meat spoilage [9,10]. Ultra-performance liquid chromatography-tandem mass spectrometry (UPLC-MS/MS), liquid chromatography-electrospray ionization-mass spectrometry (LC-ESI-MS/MS), gas chromatography-mass spectrometry (GC-MS), and gas chromatography-olfactometry-mass spectrometry (GC-O-MS) are the most commonly used analytical methods based on VOCs, but these methods are susceptible to complex food matrices and have lengthy detection times due to complicated sample preparation [11,12]. As a result, these methods do not provide efficient or instant detection.

GC-MS and gas chromatography-ion mobility spectrometry (GC-IMS) are currently leading research methods for evaluating food flavor [13]. GC-MS is a technique that separates mixed samples into individual molecules in a chromatograph and then sends those molecules to mass spectrometry to be ionized into ions and fragmented ions with varying mass-to-charge ratios. The ions and fragmented ions are then accelerated and concentrated by an electromagnetic field to create a mass spectrogram. The combination of GC and MS can efficiently separate volatile and semi-volatile compounds, but also selectively detect them, resulting in effective qualitative and quantitative compound separation. However, as the complexity and diversity of analytical components increase, conventional GC-MS cannot separate highly complex mixtures in the study of volatile compounds in complex foods.

Headspace-gas chromatography-ion mobility spectroscopy (HS-GC-IMS) is widely used for VOC identification and detection in agricultural goods. The GC-IMS system ionizes volatile gas molecules into ionized molecules, and by measuring their drift time and mobility, it can determine the associated compound type and concentration [14]. Due to its benefits of low detection limits, high sensitivity, and a relatively simple system setup, HS-GC-IMS technology has advanced quickly in the field of VOC analysis. This method is primarily used in the fields of food classification and adulteration, food odor detection, food processing process monitoring, and evaluation of aroma changes during food storage. However, it is rarely used in the detection of microbial contamination of meat and meat products.

Overall, HS-GC-IMS and GC-MS were employed to analyze the variation of characteristic volatile substances produced by chicken samples during *S. aureus* early contamination. We investigated the feasibility of HS-GC-IMS in monitoring and detected early food contamination. In practice, actions may be implemented ahead of time to prevent poultry infection, lengthen storage and shelf life, and lessen chicken industry economic loss.

## 2. Materials and Methods

### 2.1. Materials and Chemicals

Two white sticks of Shiyang chicken were obtained from Renrenle supermarket, agar and LB broth were supplied by Beijing Biotopped Technology Co., Ltd., (Beijing, China). and distilled water was purchased from Watson’s supermarket. J&K Scientific Ltd., China, supplied the 2-octanol standard.

### 2.2. Pretreatment of Chicken

The entire chicken was wrapped in crushed ice and carried to the laboratory. It was washed 3 times in sterilized stomacher bags with sterile water. The chicken was then separated and placed in a biosafety cabinet. A total of 5 g of chicken was weighed, divided into 3 equal pieces, cleaned twice in sterile water, placed in a 50 mL sterilized centrifuge tube, and frozen at −40 °C for storage.

Single colonies of *Staphylococcus aureus* ATCC 29213 were picked from the plates and inoculated with 1 mL of saline (0.85%, *w*/*w*) in a 1.5 mL centrifuge tube and diluted in a gradient to obtain a 10^−4^ concentration of *S. aureus* suspension. We added 200 L of the suspension to a poultry centrifuge tube, mixed thoroughly, and incubated it at 20 °C. The 6 tubes were removed at 0 h, 4 h, 8 h, 12 h, 24 h, and 48 h, respectively. They were stored in a freezer at −40 °C for HS-GC-IMS and HS-SPME-GC-MS tests. Samples were prepared by adding a 10^−6^ suspension concentration as above. The 0 h poultry without bacterial suspension served as a blank control (CK). Operations were performed in the BSL-2 biosafety laboratory. All biohazardous waste was sterilized at 121 °C for 30 min and then disposed of in accordance with the regulations to ensure the safety of the experiments.

### 2.3. Volatile Component Analysis by HS-GC-IMS

We transferred 500 L of solution from the poultry samples to the headspace bottle, incubated at 40 °C for 15 min with the needle at 75 °C, and aspirated 500 L of gas for detection from above the headspace bottle. In this experiment, an FC-SE-54 column was chosen with the following parameters: a column temperature of 40 °C, high-purity N_2_ as the carrier gas, a programmed flow rate of 2.00 mL/min for 2 min, and a linear increase to 100 mL/min within 20 min. The IMS drift tube temperature was set to 45 °C, and high-purity N_2_ was used as the drift gas at a flow rate of 150 mL/min.

### 2.4. HS-SPME-GC-MS Analysis

The chicken sample was washed with 5 mL sterile water, and the dilution was transferred into a 20 mL headspace bottle along with 9 μL of 2-octanol solution at a concentration of 0.1638 mg/mL (final concentration of 29.425 μg/L), plus a gasket to seal the bottle with a cap. Samples were incubated at 80 °C for 20 min, and selected 3-phase DVB/CAR/PDMS fibers (50/30 μm, Agilent Technologies, Santa Clara, CA, USA) were used to extract volatile and semi-volatile gases above the vial. SPME fibers were aged for the first time at 250 °C for 1 h. SPME fibers were stabbed into the headspace of the vial, extracted at 85 °C for 30 min, and then injected into the GC port at 250 °C for 5 min.

A 7890B GC equipped with the 5977B Quality Selection Detector (MSD, Agilent Technologies, USA) was selected for this experiment. The GC was equipped with an HP-5 MS analytical column (19091S-433UI) to separate complex compounds. The GC heating procedure was as follows: the initial temperature was 40 °C for 3 min, then increased to 70 °C at 3 °C/min, then increased to 180 °C at 5 °C/min, and finally raised to 230 °C at 10 °C/min and held for 5 min. The carrier gas was He and the flow rate was 1.6 mL/min. The mass spectrometry conditions were: at 70 eV, the m/z scanning range was 30~550, the ion source temperature was 230 °C, and the quadrupole temperature was 140 °C. All samples were performed in triplicate. Using 2-octanol as the internal standard, the relative percentage content and retention index (RI) of each compound were calculated.

### 2.5. Statistical Analysis

In this study, all results were expressed as mean ± standard deviation (SD) (n = 3). SPSS 25.0 software (SPSS Inc., Chicago, IL, USA) was employed for univariate significance difference (*p* < 0.05) and correlation coefficient calculations. Using SIMCA 14.1 (MSK Umetrics AB) for orthogonal partial least squares discriminant analysis (OPLS-DA), the value of variable important on the projection (VIP) was obtained, which was a significant indicator for evaluating metabolic marker screening [15]. We used the MetaboAnalyst 5.0 website to perform a cluster of VOCs (https://www.metaboanalyst.ca/ (accessed on 23 May 2023)). Venn diagrams were produced through the Draw Venn Diagram (http://bioinformatics.psb.ugent.be/webtools/Venn/ (accessed on 25 May 2023)) and Evenn (http://www.ehbio.com/test/venn/#/ (accessed on 25 May 2023)) websites. M-F represented the group of chicken meat inoculated with a 10^−4^ concentration of bacterial suspension (the number of bacteria in chicken meat is greater than countable), as determined by HS-SPME-GC-MS. M-S represented the group of chicken meat inoculated with a 10^−6^ concentration of bacterial suspension (the number of *S. aureus* in chicken meat is 15 CFU/g), as determined by HS-SPME-GC-MS. I-F represented the group of chicken meat inoculated with a 10^−4^ concentration of bacterial suspension as detected by HS-GC-IMS, and I-S represented the group of chicken meat inoculated with bacterial suspension of 10^−6^ concentration as detected by HS-GC-IMS.

## 3. Results and Discussion

### 3.1. Qualitative Analysis of Volatile Compounds in Chicken at Different Contamination Times

After analyzing by HS-SPME-GC-MS and HS-GC-IMS, 62 and 64 VOCs were detected in chicken samples, respectively. After removing the outliers, the data for all compounds identified in chicken meat are listed in Supplementary Data (Tables S1–S6). These substances were broadly classified into seven categories, including alcohols, aldehydes, ketones, hydrocarbons, esters, sulfur compounds, acids, and some other types of compounds. HS-SPME-GC-MS detected 7 alcohols, 13 aldehydes, 2 ketones, 16 hydrocarbons, 11 esters, 4 sulfur compounds, and 9 others. HS-GC-IMS detected 12 alcohols, 13 aldehydes, 12 ketones, 11 esters, 3 sulfur compounds, 4 acids, and 10 others. Among them, HS-GC-IMS detected seven substances with both monomers and dimmers. This is related to the concentration of volatile substances in the drift tube and their half-lives [16,17]. These seven substances are 3-methyl-1-butanol, 1-pentanol, hexanal, 2-methylbutanal, benzaldehyde, ethyl acetate, and heptan-2-one. These results are similar to Deng et al. [18].

**Figure 1:**
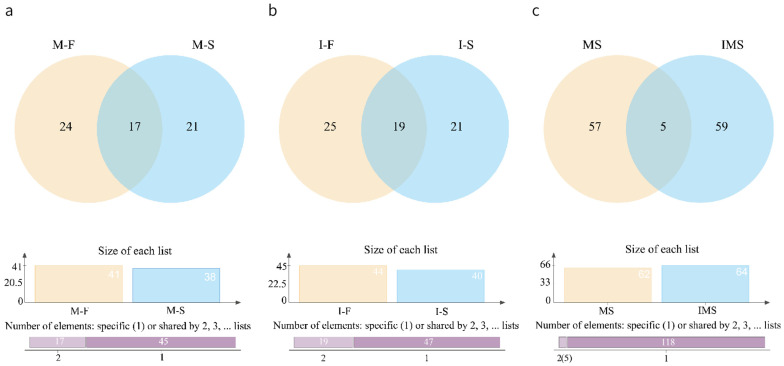
Venn diagram of VOCs detected at two concentrations using two methods; (**a**) comparison of M-F and M-S; (**b**) comparison of I-F and I-S; (**c**) comparison of HS-SPME-GC-MS and HS-GC-IMS. M-F: substances in 10^−4^ concentration of *S. aureus*-contaminated samples detected by HS-SPME-GC-MS.; M-S: substances in 10^−6^ concentration of *S. aureus*-contaminated samples detected by HS-SPME-GC-MS; I-F: substances in 10^−4^ concentration of *S. aureus*-contaminated samples detected by HS-GC-IMS; I-S: substances in 10^−6^ concentration of *S. aureus*-contaminated samples detected by HS-GC-IMS.

Venn diagrams of VOCs in chicken at two concentrations are shown in Figure 1. There were 17 substances commonly detected at two concentrations by the HS-SPME-GC-MS method. They were toluene, 2,6,10,15-tetramethylheptadecane, tetradecane, hexanal, heptanal, 1-octen-3-ol, nonanal, 3-ethyl-5-(2-ethylbutyl) octadecane, 2,6,10-trimethyltetradecane, 2,4-bis(2-methyl-2-propanyl) phenol, heptacosane, hexadecane, stearaldehyde, dimethyltetrasulfane, 2-undecenal, 3-methyl-1-butanol, and 1H-indole (Figure 1a). In M-F and M-S, 24 and 21 components were detected separately, respectively. There were 19 substances detected by the HS-GC-IMS method at both concentrations together. They were ethyl acetate, cyclohexanone, 2-methylbutanal, 3-butenenitrile, 1-pentanol, 3-hydroxy-2-butanone, hexanal, 2-hexanol, 3-methyl-1-butanol, heptan-2-one, ethyl 2-methylpropanoate, butan-2-one, 3-methylbutanal, 2-pentanone, (2E)-2-pentenal, 1-hexanol, aniline, styrene, and 2-methylpropionic acid (Figure 1b). A total of 26 and 21 volatile components were detected separately in I-F and I-S, respectively.

### 3.2. Relative Abundance Analysis of Volatile Compounds in Chicken at Different Contamination Times

#### 3.2.1. Analysis of the Relative Content of Volatile Compounds in Chicken Meat by HS-SPME-GC-MS

**Figure 2:**
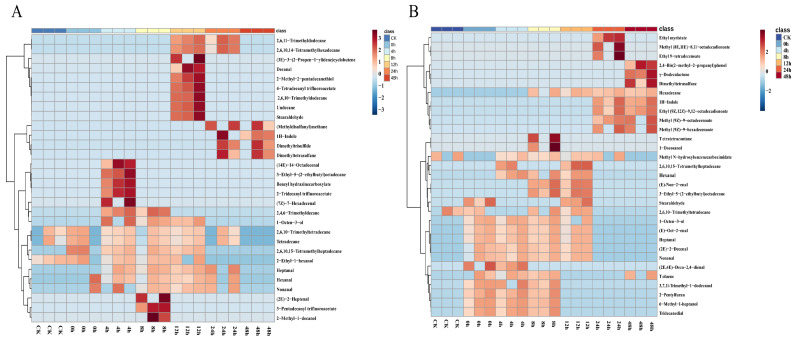
Cluster heat map of volatile components of chicken meat at different incubation times by HS-SPME-GC-MS; (**A**) substances detected at 10^−4^ concentration of *S. aureus*-contaminated samples; (**B**) substances detected at 10^−6^ concentration of *S. aureus*-contaminated samples; “CK” served as a blank control.

**Figure 3:**
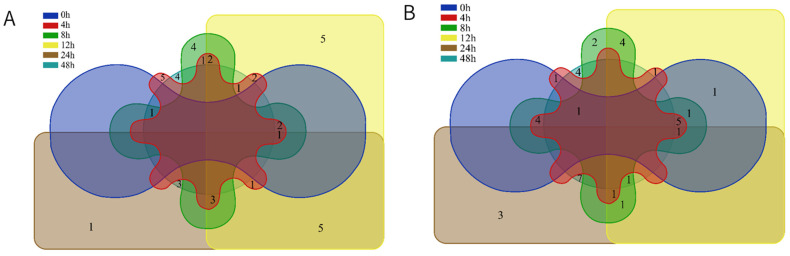
Venn diagram of six time points for volatile components of chicken meat by HS-SPME-GC-MS; (**A**) 10^−4^ concentration bacterial suspension inoculation group; (**B**) 10^−6^ concentration bacterial suspension inoculation group.

HS-SPME and GC-MS were used to analyze the volatile component variation in *S. aureus*-contaminated chicken. The changes in VOCs during early-stage contamination are shown in Figure 2. In the chicken meat of M-F, the five time points from 4–48 h separately detected five, four, five, one, and four substances, respectively (Figure 3A), and 0–24 h commonly revealed four substances, including nonanal, tetradecane, 2,6,10-trimethyltetradecane, and hexanal. In the chicken meat of M-F, CK group, and at zero hours, 2,6,10-trimethyltetradecane, tetradecane, 2,6,10,15-tetramethylheptadecane and 2-ethyl-1-hexanol were identified. At zero hours, hexanal and nonanal were detected. Three sulfur compounds ((methyldisulfanyl) methane, dimethyltrisulfane, and dimethyltetrasulfane) and 1H-indole showed an increasing trend. The content of (methyldisulfanyl) methane also showed a rising trend with time during pork storage [15]. 14-Octadecenal, 3-ethyl-5-(2-ethylbutyl) octadecane, benzyl hydrazinecarboxylate and (7Z)-7-hexadecenal appeared only at four hours. (2E)-2-heptenal, 3-pentadecanyl trifluoroacetate, and 2-methyl-1-decanol were only detected at eight hours. (3E)-3-(2-Propen-1-ylidene) cyclobutene, decanal, 2-methyl-2-pentadecanethiol, 4-tetradecanyl trifluoroacetate, 2,6,10-trimethyldodecane, undecane, and stearaldehyde only appeared at 12 h. Binding affinity for the KCSA potassium channel 3-pentadecanyl trifluoroacetate and 4-trifluoroacetoxytetradecane were reported to be present in methanolic extracts of some plants [19,20]. Due to their high binding affinity for the KcsA potassium channel, they have significant antibacterial, anti-inflammatory, and other bioactive functions [21]. They have been used as potential natural antibacterial drugs [22]. In this study, both substances appeared at 8 h and 12 h of contamination in the high-concentration contamination group and were not detected in the low concentration group. Some studies have reported that some bacteria (*Xenorhabdus* and *Photorhabdu*) produce this substance during growth and reproduction, thus competing to inhibit the growth of other cultures and microorganisms [23], but whether *S. aureus* also had this ability was not certain based on our results. Further analysis still needs to be conducted. A trend of increasing and then decreasing relative content of most alkanes was found in Figure 2A. 2,6,11-Trimethyldodecane, 2,6,10,14-tetramethylhexadecane, 2,4,6-trimethyldecane, 1-octen-3-ol, and heptanal were not detected in the CK group or at zero hours. Their contents showed a trend of first increasing and then decreasing. These five substances were likely to be the characteristic components of *S. aureus* contamination in chicken meat. The three sulfur compounds, (methyldisulfanyl) methane, dimethyltrisulfane, and dimethyltetrasulfane, started to appear at 24 h and showed an increasing trend, which was similar to Liu et al. [16]. These three sulfur compounds might also be biomarkers of early contamination of chicken meat with *S. aureus*.

In the chicken of M-S, one substance consistently observed from 0 to 24 h was 2-Undecenal, and five compounds existed from 0 to 12 h (nonanal, (E)-oct-2-enal, 1-octen-3-ol, (2E)-2-decenal, and heptanal). Two, three, and four substances were detected separately at 8 h, 24 h, and 48 h, respectively (Figure 3B). The clustering analysis of the volatile components obtained from the M-S is shown in Figure 2B and there were three trends in the relative content of substances over time: an increasing trend followed by a declining trend. The only substance detected in both the CK group and at zero hours was 2,6,10-trimethyltetradecane. The top three substances (ethyl myristate, methyl (8E,11E)-8,11-octadecadienoate, and ethyl 9-tetradecenoate) and five compounds in the middle (tetratetracontane, 1-docosanol, hexanal, (E)-non-2-enal, 3-ethyl-5-(2-ethylbutyl) octadecane) showed an increasing trend followed by a decreasing trend. The lower 10 compounds (1-octen-3-ol, (E)-oct-2-enal, heptanal, (2E)-2-decenal, nonanal, (2E,4E)-deca-2,4-dienal, 3,7,11-trimethyl-1-dodecanol, 2-pentylfuran, 6-methyl-1-heptanol, and tridecanedial) showed a decreasing trend, and the upper 8 substances (2,4-bis(2-methyl-2-propanyl) phenol, γ-dodecalactone, dimethyltetrasulfane, hexadecane, 1H-indole, ethyl (9Z,12Z)-9,12-octadecadienoate, methyl (9Z)-9-octadecenoate, and methyl (9Z)-9-hexadecenoate) showed an increasing trend.

#### 3.2.2. Analysis of the Relative Abundance of Volatile Compounds in Chicken Meat by HS-GC-IMS

**Figure 4:**
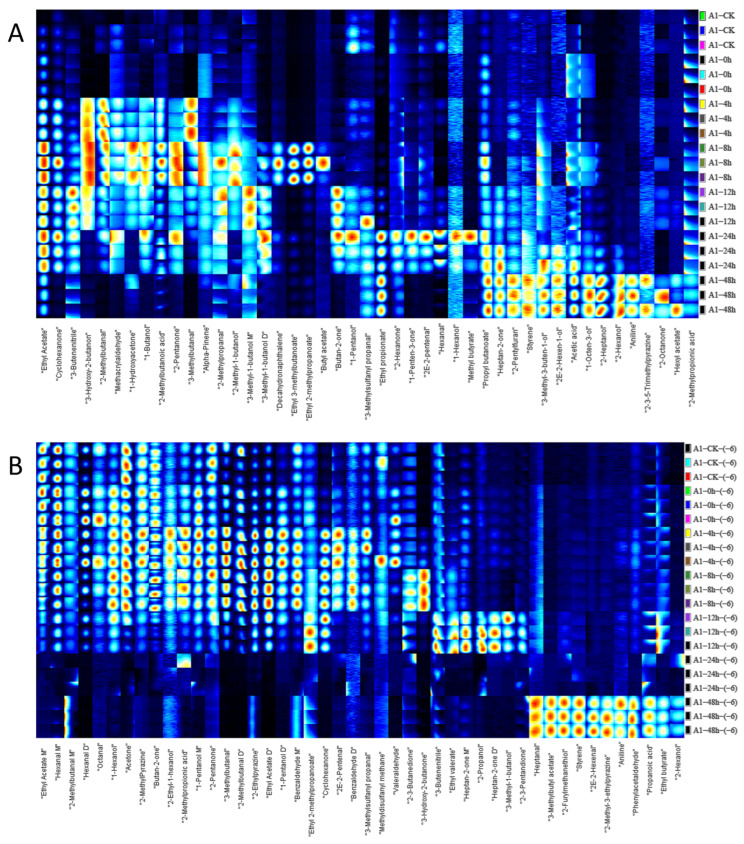
Fingerprint spectra of volatile components in chicken meat at different incubation times (from top to bottom is 0 h, 4 h, 8 h, 12 h, 24 h, 48 h) by HS-GC-IMS; (**A**) substances detected at 10^−4^ concentration of *S. aureus*-contaminated samples; (**B**) substances detected at 10^−6^ concentration of *S. aureus*-contaminated samples; (M) for monoblock and (D) for dimer.

As shown in Figure 4A, 30 substances on the left side of the I-F sample demonstrated a rising and then decreasing trend with the increase in incubation time, and 15 substances on the right side showed an increasing trend with the increase in time. Among the substances increasing and then decreasing, six substances (ethyl acetate, cyclohexanone, 3-hydroxy-2-butanone, 2-methylbutanal, 3-methylbutanal, and butan-2-one) showed significant changes and can be considered as potential characteristic VOCs. Among the substances with increasing content, the changes in 1-octen-3-ol and 2-heptanol were more pronounced than the other substances. We can also see that at zero hours, the number of detected substances was less. According to Marta Mikš-Krajnik et al. [24], this is probably because at zero hours the chicken has not yet deteriorated.

A total of 46 volatile compounds were detected in the sample of I-S. On the left side, 35 substances showed a trend of increasing and then decreasing with time, and 11 substances on the right side were increasing with time (Figure 4B). Among them, 14 substances (hexanal M, 1-hexanol, acetone, 2-methylpyrazine, butan-2-one, 1-pentanol D, 1-pentanol M, 2-pentanone, 3-methylbutanal, 2-methylbutanal D, 2-ethylpyrazine, ethyl acetate D, cyclohexanone, and 3-hydroxy-2-butanone) showed a trend of rising and then decreasing relative content over time that was more obvious among the 46 substances, and the time points of the highest content of these substances were different. The substances with increased contents were detected after 48 h. Among I-F and I-S, eight substances, including ethyl acetate D, 2-methylpyrazine, 3-methylbutanal, 3-hydroxy-2-butanone, 2-methylbutanal D, 2-furfurylthiol, 3-methylbutyl acetate, and aniline, showed consistent trends in relative abundance and are likely to be potential characteristic spoilage substances.

### 3.3. Different Metabolites Associated with Spoilage Flavour Formation in Chicken Meat

**Figure 5:**
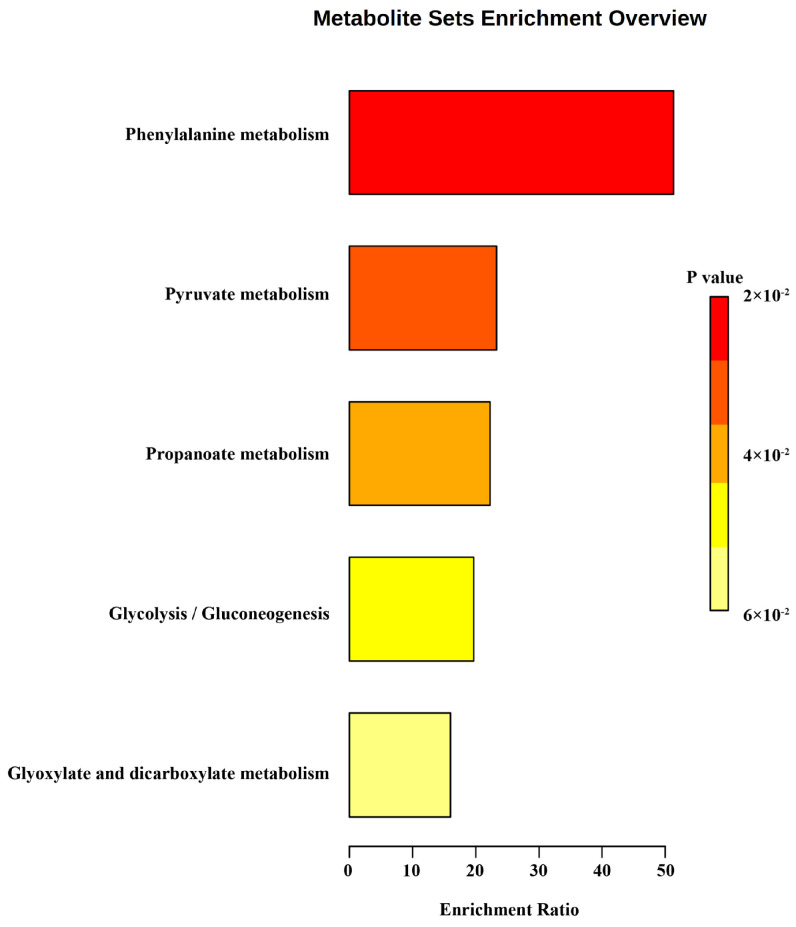
KEGG enrichment of all VOCs.

To clarify the metabolite information of *S. aureus*, all compounds detected by both methods were subjected to KEGG metabolic enrichment. These volatiles focused on phenylalanine metabolism, pyruvate metabolism, propanoate metabolism, glycolysis/gluconeogenesis, glyoxylate, and dicarboxylate metabolism (Figure 5). This indicates that *S. aureus* metabolism in chicken meat is dominated by basic metabolism. Some of the metabolites with more significant changes in relative abundance were retrieved from the KEGG database as shown in Table 1.

#### 3.3.1. Alcohols

The biosynthesis of alcohols in meat is associated with many metabolic pathways, including protein hydrolysis, amino acid metabolism, methyl ketone reduction, and the reduction of aldehydes from lipid oxidation [12,25,26]. Both methods in this study detected 1-octen-3-ol and showed similar trends. 1-Octen-3-ol has been detected in many meats [12,26,27,28,29,30], it is a major flavor component in chicken [31], and it correlates with peroxide value and thiobarbituric acid reactive substances (TBARS) [25]. Linoleic and linolenic acids might be 1-octen-3-ol precursors. The enzymatic metabolism of linolenic acid and the autoxidation of linoleic acid autoxidation produces 1-octen-3-ol [26,32]. Geeraerts et al. [33] regarded 1-octen-3-ol as a biologic for bacterial growth or chemical degradation of cooked poultry product markers and suggested that 1-octen-3-ol is a product of lipid oxidation. Therefore, 1-octen-3-ol in chicken meat may come from the oxidation of lipids or the enzymatic degradation of linoleic acid.

#### 3.3.2. Aldehydes

**Figure 6:**
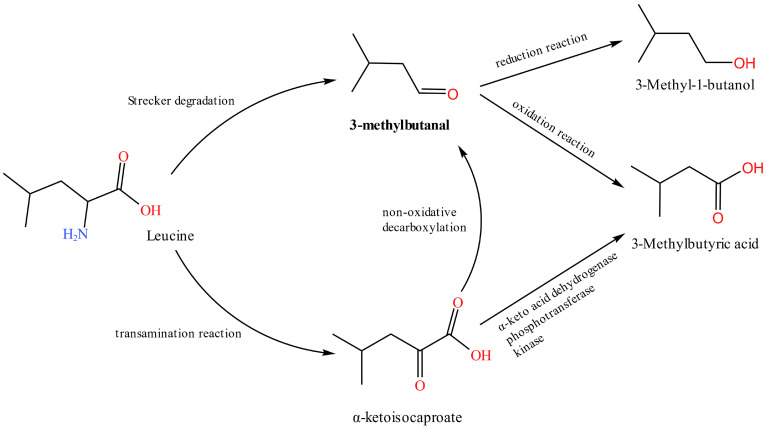
3-Methylbutanal, 3-Methyl-1-butanol, and 3-Methylbutyric acid (isovaleric acid) are produced by the catabolism of leucine, which has been found to be significantly released by *Staphylococcus aureus*.

Aldehydes are mainly derived from the hydrolysis of triglycerides and the fatty acid metabolism [13] and can also occur during Merad-induced degradation of amino acids [30]. Due to their high volatility and low threshold, aldehydes contribute significantly to the characteristic flavor of cooked chicken and dry-cured ham [12,30]. The relative levels of the three potentially characteristic aldehydes 2-methylbutanal, 3-methylbutanal, and heptanal detected in this study all showed a trend of increasing and decreasing over time. The first increase was mainly due to the oxidation of lipids in chicken meat, and the later decrease in content may be because of the rapid oxidation of these aldehydes to acids and subsequent esterification by ethanol [34]. KEGG metabolite searches revealed that the upstream substances of 2-methylbutanal and 3-methylbutanal are 3-methyl-2-oxopentanoic acid and 3-methyl-1-butanol, respectively. 3-Methyl-1-butanol is converted to 3-methylbutanal by 3-methylbutanal reductase. 3-Methylbutanal and 2-methylbutanal are also amino acid modifiers and intermediates for the formation of many esters and branched ketones, and 3-methylbutanal is the fingerprint compound of *S. aureus* [35]. 3-Methylbutanal can also be formed by the leucine metabolism (Figure 6). In the metabolism of *S. aureus*, 3-methylbutanal can be reduced to 3-methyl-1-butanol by alcohol dehydrogenase or oxidized to 3-methylbutyric acid by aldehyde dehydrogenase [35]. According to Hu et al. [35], 3-methylbutanal is an aldehyde specifically produced by *S. aureus*. The concentration of this substance increased significantly early in the culture, in agreement with this study. Heptanal is mainly produced by the oxidation of arachidonic acid [12]. Heptanal contributes to fatty and green herbal odors but is very unpleasant when its concentration exceeds the odor threshold [28,29]. 3-Methylbutanal in chicken meat in this experiment may be obtained by 3-methyl-1-butanol esterification or the leucine metabolism, which is later oxidized into 3-methylbutyric acid or reduced into 3-methyl-1-butanol. Heptanal may be produced by the oxidation of arachidonic acid. It has a negative impact on chicken meat flavor when its odor threshold is exceeded.

#### 3.3.3. Esters

There are two pathways for ester synthesis: spontaneous formation (chemical) and enzymatic synthesis. Three types of reactions are included: esterification, alcoholysis, and acidysis, where esterification is the most common pathway for the microbial synthesis of esters [36]. Esters in meat are mainly formed by the esterification of alcohols and carboxylic acids [37]. The relative abundance of ethyl acetate increased and then decreased in both I-F and I-S, similar to Chen et al. Ethyl acetate is a useful chemical indicator for assessing meat freshness [32], and it is generally believed that esterification of acetic acid and ethanol yields ethyl acetate [38]. Meanwhile, in the KEGG database, ethyl acetate involves reaction R12515, which is the reaction between acetate and ethanol to produce ethyl acetate. 3-Methylbutyl acetate is formed by the esterification reaction between reduced acetic acid and 3-methyl-1-butanol [39]. In KEGG, 3-methylbutyl acetate was included in reaction R12516, in which the esterification reaction of 3-methyl-1-butanol and ethanol produced this substance. Therefore, we presume that ethyl acetate in this study was derived from the esterification reaction between acetate and ethanol, and 3-methylbutyl acetate was produced by the esterification reaction between 3-methyl-1-butanol and acetate.

#### 3.3.4. Ketones

Ketones are mainly produced by the oxidation of unsaturated fatty acids, the Melad reaction, and the oxidation of alcohols [26,29,40,41,42]. Methyl ketones can be formed by β-keto acid decarboxylation or β-oxidation of saturated fatty acids. They are precursors of the fat aroma in cooked meat [12]. Both methods detected 3-hydroxy-2-butanone and cyclohexanone in chicken meat and showed the same trends. 3-hydroxy-2-butanone is formed from pyruvate (pyruvate) during the low pH fermentation of glucose by *S. aureus* [43]. 3-Hydroxy-2-butanone is related to the metabolic activity of certain microorganisms (e.g., *Brochothrix thermosphacta* and *Pseudomonas* spp.) [41] and causes a significant increase in ketones during bacon storage [18]. Ketones usually have floral and creamy flavors, but their odor threshold is much higher than the threshold corresponding to isomeric aldehydes and has a lower effect on chicken flavor [26,29]. 2,6,10,15-Tetramethylheptadecane involves four reactions in KEGG, converting to a ketone 2,3-buranedione and an ester acetaldehyde. Cyclohexanone in KEGG is involved in map00930 (caprolactam degradation), map01120 (Microbial metabolism in diverse environments) and map01220 (degradation of aromatic compounds). Three pathways, including five reactions (R02229, R02231, R02232, R02233, R02234) in the R02229 cyclohexanone react with NADH to produce an alcohol: cyclohexanol. Therefore, ketones are presumed to be produced by the oxidation of unsaturated fatty acids or alcohols and then react to produce other types of organic substances.

#### 3.3.5. Sulfur Compound

The metabolism and secondary metabolic reactions of nitrogenous compounds and amino acids lead to sulfur compound formation [15]. Sulfur compounds are typical compounds indicative of pork spoilage under low oxygen conditions [11]. The precursors of dimethyltrisulfane (DMTS) are methionine and sulfides methanethiol (MeSH), and sulfur compounds are the most prospective markers of volatile spoilage in pork [15]. According to Schulz et al. [43], DMTS is produced in bacteria by two pathways: ascorbate and transition-metal ions mediating the production of hydrogen sulfide and methanethiol by a rapid autooxidation reaction and disproportionation of dimethyl disulfide to generate DMTS. There was a significant increase in the abundance of DMTS in I-S chicken at four hours and eight hours, which may be produced by the disproportionation of methionine or dimethyl disulfide, and DMTS is probably a marker of chicken spoilage.

### 3.4. Identification of Chicken Spoilage at Different Times Based on Chemometrics

#### 3.4.1. The Orthogonal Partial Least Squares Discrimination Analysis of VOCs in Chicken at Different Contamination Times

**Figure 7:**
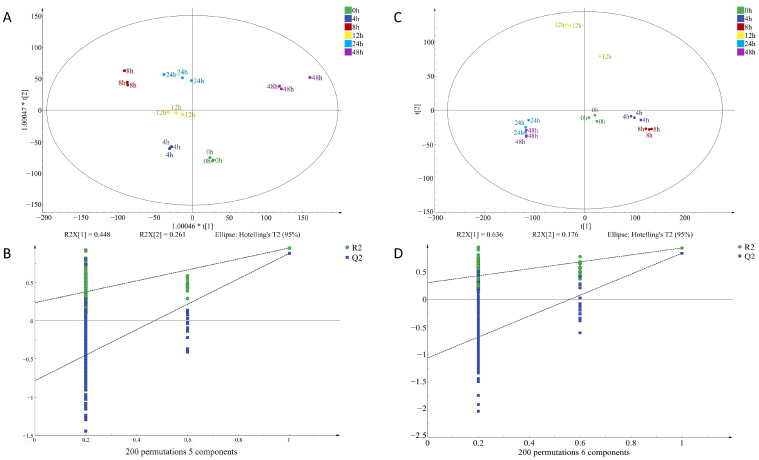
OPLS-DA of volatile organic compounds in chicken meat contaminated with *Staphylococcus aureus* at different incubation times by HS-GC-IMS; (**A**) factor loading diagram at 10^−4^ concentration of *S. aureus*-contaminated samples; (**B**) displacement test results at 10^−4^ concentration of *S. aureus*-contaminated samples; (**C**) factor loading diagram at 10^−6^ concentration of *S. aureus*-contaminated samples; (**D**) displacement test results at 10^−6^ concentration of *S. aureus*-contaminated samples.

**Figure 8:**
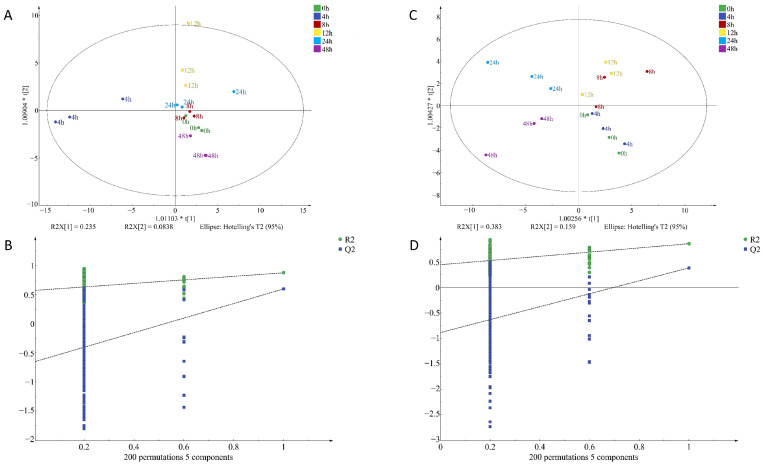
OPLS-DA of volatile organic compounds in chicken meat contaminated with *Staphylococcus aureus* at different incubation times by HS-SPME-GC-MS; (**A**) factor loading diagram at 10^−4^ concentration of *S. aureus*-contaminated samples; (**B**) displacement test results at 10^−4^ concentration of *S. aureus*-contaminated samples; (**C**) factor loading diagram at 10^−6^ concentration of *S. aureus*-contaminated samples; (**D**) displacement test results at 10^−6^ concentration of *S. aureus*-contaminated samples.

To identify potential volatile compounds in raw chicken meat contaminated with *S. aureus* at different times, a supervised orthogonal partial least squares discrimination analysis, (OPLS-DA) was performed on the signal intensity of volatile flavor substances at two concentrations (Figure 7 and Figure 8). OPLS-DA can remove the signals related to the X and Y axes and model the relationship between substance expression and the sample category using partial least squares regression and also effectively separate samples and predict sample categories. Figure 7A indicates that the VOCs at every time point in I-F were clearly distinguished, illustrating that the model had a significant classification effect at this inoculum amount, and the difference between groups was obvious. Figure 7D clearly shows the difference in VOCs between the four time points of 0–12 h, but there was some overlap between the 24 h and 48 h flavors, and the classification effect of the model in I-S was still relatively significant. Figure 8A indicates that the VOCs at the six time points were differentiated, but the components at zero hours and eight hours were slightly overlapping. The model also exhibited a good classification effect in M-F, and the difference between groups was relatively small. Figure 8D was the classification of VOCs at six time points in M-S. The substances at six time points were distinguished more obviously, and the classification effect of this model in M-S was more significant, with clear differences between groups.

Q^2^ represents the prediction ability of the model and R^2^ represents the explanation ability of the model. The closer the values of Q^2^ and R^2^ are to one in theory, the better the model fit is. In this study, R_X_^2^_c_ = 0.963, R_Y_^2^_c_ = 0.979, Q^2^_c_ = 0.884; R_X_^2^_f_ = 0.986, R_Y_^2^_f_ = 0.978, Q^2^_f_ = 0.933 (Figure 7); R_X_^2^_c_ = 0.876, R_Y_^2^_c_ = 0.853, Q^2^_c_ = 0.57; R_X_^2^_f_ = 0.896, R_Y_^2^_f_ = 0.808, Q^2^_f_ = 0.43 (Figure 8). This indicated that the stability and predictive ability of the constructed model were good and VOC composition in chicken meat at different incubation times.

Variable importance in the projection (VIP) quantifies the contribution of each component to the classification. Among them, VOCs with VIP values greater than one can be regarded as potential characteristic markers. Combining the two methods and concentrations analysis, the potential characteristic markers are shown in Table 2. The potential characteristic markers included 3 acids, 5 alcohols, 5 aldehydes, 6 esters, 11 hydrocarbons, 3 ketones, and 3 others. The characteristic markers shared by GC-MS at two concentration gradients included 2,6,10,15-tetramethylheptadecane, and the characteristic markers shared by GC-IMS at two concentration gradients were ethyl acetate, hexanal, 2-methylbutanal, butan-2-one, 3-hydroxy-2-butanone, 3-methylbutanal, and cyclohexanone.

Based on the principle that VIP > 1 was detected in both concentrations of the same method or VIP > 1 was detected in both methods, 2,6,10,15-tetramethylheptadecane, ethyl acetate, hexanal, 2-methylbutanal, butan-2-one, 3-hydroxy-2-butanone, 3-methylbutanal, and cyclohexanone were determined as characteristic biomarkers for early contamination of chicken meat with *S. aureus*.

As a substance with a VIP > 1 in M-F or I-F was detectable in both concentration gradients of the same method, and the substance was detected in both methods and the VIP was >1 in one of the methods, 1-Octen-3-ol, tetradecane, 2-hexanol, 3-methyl-1-butanol, and ethyl 2-methylpropanoate were identified as potential characteristic biomarkers of early contamination of chicken meat with *S. aureus*.

#### 3.4.2. Comparison of HS-SPME-GC-MS and HS-GC-IMS

**Figure 9:**
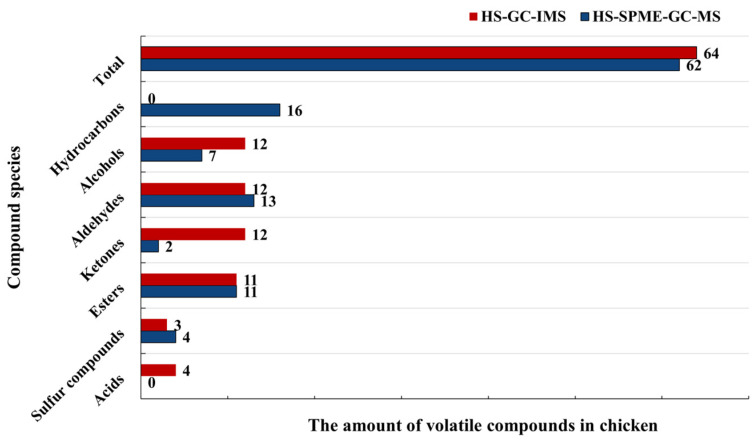
Comparison of the amount of volatile flavor compounds detected by HS-SPME-GC-MS and HS-GC-IMS.

Both HS-SPME-GC-MS and HS-GC-IMS can distinguish volatiles in chicken meat at different treatment times. However, HS-GC-IMS provided a more visual representation of the differences in volatile components in chicken meat. As shown in Figure 9, the number of alcohols and ketones identified by HS-GC-IMS was much higher than those determined by HS-SPME-GC-MS, while the number of sulfur compounds identified by HS-SPME-GC-MS was higher than those identified by HS-GC-IMS. Aldehydes and esters were detected in equal amounts by both methods. In addition, HS-GC-IMS detected 6 acids that were not detected by HS-SPME-GC-MS, and HS-SPME-GC-MS detected 16 hydrocarbons that were not detected by HS-GC-IMS. HS-GC-IMS was more sensitive to trace small molecule volatile compounds, which exhibits relatively low boiling points [13]. The combination of HS-SPME-GC-IMS and HS-GC-MS allowed for more comprehensive detection of volatiles in contaminated chicken meat.

Among all the compounds detected, five substances were detected simultaneously by both methods (Figure 1c). They were 2-octanone, 1-octen-3-ol, hexanal, heptanal, and cyclohexanone. The range of substances analyzed by HS-SPME-GC-MS and HS-GC-IMS was different for mainly two reasons: firstly, SPME injection has a greater SPME enrichment process than direct headspace injection, which is generally used for the determination of very volatile components. The differences in separation reasons and extraction methods between the two methods lead to different detection profiles. Secondly, there are differences in the detection principles of the two methods: GC-MS determines the ions mainly based on *m/z*, while GC-IMS obtains information from the product ions, so there are differences in the sensitivity of the two instruments. Among them, GC-IMS is more sensitive, with lower detection limits for short-chain volatile components and higher sensitivity for acids and furans, while GC-MS is more sensitive for long-chain volatile components [41].

## 4. Conclusions

Based on HS-SPME-GC-MS, HS-GC-IMS, and KEGG analyses, 2,6,10,15-tetramethylheptadecane, ethyl acetate, hexanal, 2-methylbutanal, butan-2-one, 3-hydroxy-2-butanone, 3-methylbutanal, and cyclohexanone were identified as characteristic biomarkers during *S. aureus* early-stage contamination (within 48 h) in chicken. 1-octen-3-ol, tetradecane, 2-hexanol, 3-methyl-1-butanol, and ethyl 2-methylpropanoate are potential characteristic biomarkers. The two methods had distinct benefits for various substances. The combination of the two methods allowed for a more precise determination of food decomposition processes. The signature volatiles identified in this study could serve as a foundation for future VOC-based rapid detection of contaminating spoilage bacteria in chicken meat. Nonetheless, there are some limitations to this research. The applicability of the detected signature volatiles to other poultry products requires further investigation. Secondly, some of the signature volatiles were discovered for the first time, and it will be necessary to investigate their specific transformation pathways in conjunction with future in-depth experiments. The application of early detection methods for contaminated pathogens based on VOCs will expand and become more convenient based on the findings of the current study and future research.

## Figures and Tables

**Table 1:** KEGG database information for differential volatile organic compounds.

Super Class	Metabolite	KEGG ID	Upstream Products	Downstream Products	Reaction	Pathway
Alcohols	1-Octen-3-ol	C14272	−	−	−	−
	1-Pentanol	C16834	C12650;	−	R08220;	−
Aldehydes	2-Methylbutanal	C02223	C00671;	−	R03894;	−
	3-Methylbutanal	C07329	C07328;	−	R05685; R05686;	−
	Heptanal	C14390	−	−	−	−
Esters	Ethyl Acetate	C00849	C00469;	C00033; C00469;	R11957; R12515;	−
	3-Methylbutyl acetate	C12296	−	C07328; C00033;	R12516	−
Hydrocarbons	Styrene	C07083	C07111; C00423;	C07084; C20782;	R05417; R05424; R05488; R11070;	map00642; map01220; map01120; map01100;
Ketones	3-Hydroxy-2-butanone	C00466	C03046;	C00741; C00084;	R02343; R02344; R02345; R09524;	−
	2-Pentanone	C01949	C02445;	−	R03781	−
	Ethyl methyl ketone	C02845	C18796;	−	R09358	−
	Cyclohexanone	C00414	C04316; C00571;	C00854; C01880; C02395;	R02229; R02231; R02232; R02233; R02234;	map00930; map01120; map01220;
	Acetone	C00207	C00164; C02659; C06551; C06559; C09276; C00448; C19707; C16143; C01845;	C01845; C01995; C00164; C17530;	R01366; R01550; R01553; R03796; R05564; R05568; R05735; R08207; R08543; R09002; R09487; R10703; R10704;	map00650; map01100;
Sulfur compounds	Dimethyltrisulfane	C08372	−	−	−	−

“−” for not related.

**Table 2:** Volatile organic compounds with a VIP value greater than one.

Super Classs	Compounds	M-F	M-S	I-F	I-S	M and I
Acids	2-Methylbutanoic acid	−	−	+	−	−
	Acetic acid	−	−	+	−	−
	2-Heptanol	−	−	−	+	−
Alcohols	3-Methyl-1-butanol	−	−	+	−	−
	2-Hexanol	−	−	+	−	−
	1-Octen-3-ol	−	−	+	−	−
	2-Propanol	−	−	+	−	−
	Hexanal	−	−	−	+	−
Aldehydes	2-Methylbutanal	−	+	+	+	+
	3-Methylbutanal	−	−	+	+	−
	2-Undecenal	−	−	+	+	−
	Stearaldehyde	−	+	−	−	−
	Ethyl Acetate	−	+	−	−	−
Esters	Hexyl acetate	−	−	+	+	-
	Ethyl 3-methylbutanoate	−	−	+	−	−
	Ethyl propanoate	−	−	+	−	−
	Ethyl 2-methylpropanoate	−	−	+	−	−
	Propyl bytanoate	−	−	+	−	−
	Decahydronaphthalene	−	−	+	−	−
Hydrocarbons	3-Butenenitrile	−	−	+	−	−
	(14E)-14-Octadecenal	−	−	−	+	−
	2,6,11-Trimethyldodecane	+	−	−	−	−
	2,6,10,15-Tetramethylheptadecane	+	−	−	−	−
	Tetradecane	+	+	−	−	−
	Heptacosane	+	−	−	−	−
	Tetratetracontane	−	+	−	−	−
	Toluene	−	+	−	−	−
	2,6,10-Trimethyltetradecane	−	+	−	−	−
	Hexadecane	−	+	−	−	−
	Butan-2-one	−	+	−	−	−
Ketones	3-Hydroxy-2-butanone	−	−	+	+	−
	Cyclohexanone	−	−	+	+	−
	2-Heptanone	−	−	+	+	−
	Acetone	−	−	−	+	−
Others	2-Ethylpyrazine	−	−	−	+	−
	2-Tridecanyl trifluoroacetate	+	−	−	−	−
	Methyl N-hydroxybenzenecarboximidate	−	+	−	−	−

“−” for not related. “+” for detected.

## Data Availability

The datasets generated for this study are available on request to the corresponding author.

## Supplementary Materials

The following supporting information can be downloaded at: https://www.mdpi.com/article/10.3390/foods12142782/s1. Table S1: List of metabolites identified by HS-SPME-GC-MS in chickens stored for 0–48 h. Table S2: List of metabolites identified by HS-GC-IMS in chickens stored for 0–48 h. Table S3: Change in relative peak area of volatile compounds in chicken during storage (mean ± SD) and correlation analysis with storage hours. (GC-MS 10^−4^ *Staphylococcus aureus* suspension). Table S4: Change in relative peak area of volatile compounds in chicken during storage (mean ± SD) and correlation analysis with storage hours (GC-MS 10^−6^ *Staphylococcus aureus* suspension). Table S5: Signal intensity of volatiles detected in GC-IMS (10^−4^ *Staphylococcus aureus* suspension). Table S6: Signal intensity of volatiles detected in GC-IMS (10^−6^ *Staphylococcus aureus* suspension).

## Abbreviations

HS-SPME-GC-MS	Headspace solid-phase microextraction coupled with gas chromatography-mass spectrometry.
HS-GC-IMS	Headspace gas chromatography-ion mobility spectrometry.
VOCs	Volatile organic compounds.
OPLS-DA	Orthogonal partial least squares discriminant analysis.
UPLC-MS/MS	Ultra performance liquid chromatography-tandem mass spectrometry.
LC-ESI-MS/MS	Liquid chromatography-electrospray ionization-mass spectrometry.
M-F	Substances in 10^−4^ concentration of *S. aureus*-contaminated samples detected by HS-SPME-GC-MS.
M-S	Substances in 10^−6^ concentration of *S. aureus*-contaminated samples detected by HS-SPME-GC-MS.
I-F	Substances in 10^−4^ concentration of *S. aureus*-contaminated samples detected by HS-GC-IMS.
I-S	Substances in 10^−6^ concentration of *S. aureus*-contaminated samples detected by HS-GC-IMS.
MeSH	Methionine and sulfides methanethiol.
DMTS	Dimethyltrisulfane.
M and I	HS-SPME-GC-MS and HS-GC-IMS (both methods).
