# Supplementary material for: Analysis of Volatile Markers and Their Biotransformation in Raw Chicken during Staphylococcus aureus Early Contamination
Source: Foods. 2023 Jul 21;12(14):2782. doi: 10.3390/foods12142782 (PMC10379977; doi:10.3390/foods12142782)
Supplement: Supplementary file 1 [file foods-12-02782-s001.zip › foods-2471175-supplementary.pdf]

Table S1. List of metabolites identified by HS-SPME-GC-MS in chickens stored for 0-48 hours.

Super class	Metabolite	RT[1]	RT[2]	CAS	Formula
Alcohols	2-Ethyl-1-hexanol	15.961	–	104-76-7	$C_8H_{18}O$
	1-Octen-3-ol	13.866	13.854	3391-86-4	$C_8H_{16}O$
	2-Methyl-1-decanol	17.148	–	18675-24-6	$C_{11}H_{24}O$
	3-Methyl-1-butanol	4.381	4.47	123-51-3	$C_5H_{12}O$
	6-Methyl-1-heptanol	–	17.712	1653-40-3	$C_8H_{18}O$
	3,7,11-Trimethyl-1-dodecanol	–	26.295	6750-34-1	$C_{15}H_{32}O$
	1-Docosanol	–	34.984	661-19-8	$C_{22}H_{46}O$
Sulfur compounds	2-Methyl-2-pentadecanethiol	24.983	–	25360-09-2	$C_{16}H_{34}S$
	(Methyldisulfanyl)methane	4.602	–	624-92-0	$C_2H_6S_2$
	Dimethyltrisulfane	13.106	–	3658-80-8	$C_2H_6S_3$
	Dimethyltetrasulfane	22.431	22.442	5756-24-1	$C_2H_6S_4$
Aldehydes	Hexanal	6.162	6.162	66-25-1	$C_6H_{12}O$
	Heptanal	10.144	10.132	111-71-7	$C_7H_{14}O$
	Nonanal	18.757	18.733	124-19-6	$C_9H_{18}O$
	(7Z)-7-Hexadecenal	26.728	–	56797-40-1	$C_{16}H_{30}O$
	(14E)-14-Octadecenal	36.901	–	56554-89-3	$C_{18}H_{34}O$
	(2E)-2-Heptenal	12.78	–	18829-55-5	$C_7H_{12}O$
	Decanal	22.116	–	112-31-2	$C_{10}H_{20}O$
	Stearaldehyde	36.913	36.895	638-66-4	$C_{18}H_{36}O$
	2-Undecenal	26.656	26.668	2463-77-6	$C_{11}H_{20}O$
	(E)-Oct-2-enal	–	17.101	2548-87-0	$C_8H_{14}O$
	(2E)-2-Decenal	–	23.831	3913-81-3	$C_{10}H_{18}O$
	(2E,4E)-Deca-2,4-dienal	–	25.446	2363-88-4	$C_{10}H_{16}O$
	(E)-Non-2-enal	–	20.709	18829-56-6	$C_9H_{16}O$
Ketones	Cyclohexanone	9.699	–	108-94-1	$C_6H_{10}O$
	2-Octanone	14.264	–	111-13-7	$C_8H_{16}O$
Hydrocarbons	Toluene	5.159	5.147	108-88-3	C_7H_8
	2,6,10,15-Tetramethylheptadecane	24.858	24.223	54833-48-6	$C_{21}H_{44}$
	Tetradecane	27.517	27.493	629-59-4	$C_{14}H_{30}$
	2,4,6-Trimethyldecane	16.946	–	62108-27-4	$C_{13}H_{28}$
	Dodecane	21.873	–	112-40-3	$C_{12}H_{26}$
	3-Ethyl-5-(2-ethylbutyl)octadecane	24.829	29.066	55282-12-7	$C_{26}H_{54}$
	2,6,10-Trimethyltetradecane	29.784	29.897	14905-56-7	$C_{17}H_{36}$
	Heptacosane	34.628	34.592	593-49-7	$C_{27}H_{56}$
	(3E)-3-(2-Propen-1-ylidene)cyclobutene	5.176	–	52097-85-5	C_7H_8
	Undecane	16.94	–	1120-21-4	$C_{11}H_{24}$
	2,6,10-Trimethyldodecane	17.148	–	3891-98-3	$C_{15}H_{32}$
	2,6,11-Trimethyldodecane	24.247	–	31295-56-4	$C_{15}H_{32}$
	Hexadecane	32.384	32.36	629-73-2	$C_{16}H_{32}$
	2,6,10,14-Tetramethylhexadecane	34.835	–	638-36-8	$C_{20}H_{42}$
	Tridecanedial	–	24.823	63521-76-6	$C_{13}H_{24}O_2$
	Tetratetracontane	–	33.458	7098-22-8	$C_{44}H_{90}$

Esters	Benzyl hydrazinecarboxylate	5.165	–	5331-43-1	$C_8H_{10}N_2O_2$
	Ethyl 9-tetradecenoate	–	36.272	24880-50-0	$C_{16}H_{30}O_2$
	Ethyl myristate	–	36.485	124-06-1	$C_{16}H_{32}O_2$
	Methyl (9Z)-9-hexadecenoate	–	38.195	1120-25-8	$C_{17}H_{32}O_2$
	Methyl (8E,11E)-8,11-octadecadienoate	–	40.539	56599-58-7	$C_{19}H_{34}O_2$
	Methyl (9Z)-9-octadecenoate	–	40.599	112-62-9	$C_{19}H_{36}O_2$
	Ethyl (9Z,12Z)-9,12-octadecadienoate	–	41.382	544-35-4	$C_{20}H_{36}O_2$
	γ-Dodecalactone	–	34.367	148051	$C_{12}H_{22}O_2$
	Ethyl 9-hexadecenoate	–	39.091	56219-10-4	$C_{18}H_{34}O_2$
	Ethyl palmitate	–	39.34	628-97-7	$C_{18}H_{36}O_2$
	Ethyl (9Z)-9-octadecenoate	–	41.448	111-62-6	$C_{20}H_{38}O_2$
Others	2-Tridecanyl trifluoroacetate	17.142	–		$C_{15}H_{27}F_3O_2$
	2,4-Bis(2-methyl-2-propanyl)phenol	30.497	30.461	96-76-4	$C_{14}H_{22}O$
	3-Pentadecanyl trifluoroacetate	17.748	–		$C_{17}H_{31}F_3O_2$
	2-Bromooctadecanal	26.734	–	56599-95-2	$C_{18}H_{35}BrO$
	4-Tetradecanyl trifluoroacetate	17.759	–		$C_{16}H_{29}F_3O_2$
	2-Methyl-1-butanamine	4.049	–	96-15-1	$C_5H_{13}N$
	1H-Indole	25.054	25.22	120-72-9	C_8H_7N
	Methyl N-hydroxybenzenecarboximidate	–	11.284	67160-14-9	$C_8H_9NO_2$
	2-Pentylfuran	–	14.228	3777-69-3	$C_9H_{14}O$

Denotes: 1: Substances detected at 10^{-4} concentration *S. aureus* contaminated samples; 2: Substances detected at 10^{-6} concentration *S. aureus* contaminated samples; "–" for not detected.

Table S2. List of metabolites identified by HS-GC-IMS in chickens stored for 0-48 hours.

Super class	−4	−6	VOCs	MW	CAS	Formula
Alcohols	+	+	1-Pentanol	88.15	71-41-0	$C_5H_{12}O$
	+	−	1-Octen-3-ol	128.21	3391-86-4	$C_8H_{16}O$
	+	−	1-Butanol	74.12	71-36-3	$C_4H_{10}O$
	+	−	3-Methyl-3-buten-1-ol	86.13	763-32-6	$C_5H_{10}O$
	+	+	2-Hexanol	102.17	626-93-7	$C_6H_{14}O$
	+	+	3-Methyl-1-butanol	88.15	30899-19-5	$C_5H_{12}O$
	+	−	2-Methyl-1-butanol	88.15	34713-94-5	$C_5H_{12}O$
	+	−	2-Heptanol	116.2	543-49-7	$C_7H_{16}O$
	+	+	1-Hexanol	102.17	111-27-3	$C_6H_{14}O$
	+	–	(2E)-2-Hexen-1-ol	100.16	928-95-0	$C_6H_{12}O$
	−	+	2-Propanol	60.1	67-63-0	C_3H_8O
	−	+	2-Ethyl-1-hexanol	130.23	104-76-7	$C_8H_{18}O$
Sulfur compounds	−	+	3-(Methylsulfanyl) propanal	104.17	3268-49-3	C_4H_8OS
	−	+	(Methyldisulfanyl) methane	94.2	624-92-0	$C_2H_6S_2$
	−	+	2-Furylmethanethiol	114.17	98-02-2	C_5H_6OS
Aldehydes	+	–	Methacrylaldehyde	70.09	78-85-3	C_4H_6O
	+	+	2-Methylbutanal	86.13	96-17-3	$C_5H_{10}O$
	+	+	Hexanal	100.16	66-25-1	$C_6H_{12}O$
	+	–	2-Methylpropanal	72.11	78-84-2	C_4H_8O
	+	+	3-Methylbutanal	86.13	590-86-3	$C_5H_{10}O$
	+	+	*(2E)*-2-Pentenal	84.12	1576-87-0	C_5H_8O
	−	+	Valeraldehyde	86.13	110-62-3	$C_5H_{10}O$
	−	+	Benzaldehyde	106.12	100-52-7	C_7H_6O
	−	+	Octanal	128.21	124-13-0	$C_8H_{16}O$
	−	+	Phenylacetaldehyde	120.15	122-78-1	C_8H_8O
	−	+	Heptanal	114.19	111-71-7	$C_7H_{14}O$
	−	+	*(2E)*-2-Hexenal	98.14	6728-26-3	$C_6H_{10}O$
Acids	+	-	2-Methylbutanoic acid	102.13	600-07-7	$C_5H_{10}O_2$
	+	+	2-Methylpropionic acid	88.11	79-31-2	$C_4H_8O_2$
	+	–	Acetic acid	60.05	64-19-7	$C_2H_4O_2$
	–	+	Propionic acid	74.08	79-09-4	$C_3H_6O_2$
Ketones	+	+	Cyclohexanone	98.14	108-94-1	$C_6H_{10}O$
	+	+	3-Hydroxy-2-butanone	88.11	513-86-0	$C_4H_8O_2$
	+	+	Heptan-2-one	114.19	110-43-0	$C_7H_{14}O$
	+	–	2-Octanone	128.21	111-13-7	$C_8H_{16}O$
	+	+	Butan-2-one	72.11	78-93-3	C_4H_8O
	+	−	1-Hydroxyacetone	74.08	116-09-6	$C_3H_6O_2$
	+	−	1-Penten-3-one	84.12	1629-58-9	C_5H_8O
	–	+	Acetone	210.1388	5949-29-1	$C_6H_{10}O_8$
	+	+	2-Pentanone	86.13	107-87-9	$C_5H_{10}O$

	+	−	2-Hexanone	100.16	591-78-6	$C_6H_{12}O$
	−	+	Biacetyl	86.09	431-03-8	$C_4H_6O_2$
	−	+	2,3-Pentanedione	100.12	123-54-6	$C_5H_8O_2$
Esters	+	+	Ethyl acetate	88.11	141-78-6	$C_4H_8O_2$
	+	−	Ethyl propionate	102.13	105-37-3	$C_5H_{10}O_2$
	+	−	propyl butanoate	130.18	105-66-8	$C_7H_{14}O_2$
	+	−	Ethyl 3-methylbutanoate	130.18	108-64-5	$C_7H_{14}O_2$
	+	+	Ethyl 2-methylpropanoate	116.16	97-62-1	$C_6H_{12}O_2$
	+	−	Butyl acetate	116.16	123-86-4	$C_6H_{12}O_2$
	+	−	Methyl butyrate	102.13	623-42-7	$C_5H_{10}O_2$
	+	−	Hexyl acetate	144.21	142-92-7	$C_8H_{16}O_2$
	−	+	Ethyl valerate	130.18	539-82-2	$C_7H_{14}O_2$
	−	+	3-Methylbutyl acetate	130.18	123-92-2	$C_7H_{14}O_2$
	−	+	Ethyl butyrate	116.16	105-54-4	$C_6H_{12}O_2$
Others	+	+	Aniline	93.13	62-53-3	C_6H_7N
	+	−	2,3,5-Trimethylpyrazine	122.17	14667-55-1	$C_7H_{10}N_2$
	−	+	2-Ethylpyrazine	108.14	13925-00-3	$C_6H_8N_2$
	−	+	2-Methylpyrazine	94.11	109-08-0	$C_5H_6N_2$
	−	+	2-Ethyl-3-methylpyrazine	122.17	15707-23-0	$C_7H_{10}N_2$
	+	−	2-Pentylfuran	138.21	3777-69-3	$C_9H_{14}O$
	+	−	Alpha-Pinene	136.23	2437-95-8	$C_{10}H_{16}$
	+	+	3-Butenenitrile	67.09	109-75-1	C_4H_5N
	+	−	Decahydronaphthalene	138.25	91-17-8	$C_{10}H_{18}$
	+	+	Styrene	104.15	100-42-5	C_8H_8

Denotes: −4: Substances detected at 10^{-4} concentration *S. aureus* contaminated samples; −6: Substances detected at 10^{-4} concentration *S. aureus* contaminated samples; "+" for detected; "−" for not detected.

Table S3. Change of relative peak area of volatile compounds in chicken during storage (mean ± SD) and correlation analysis with storage hours. (GC-MS 10^{-4} *Staphylococcus aureus* suspension)

Metabolite	CK	0h	4h	8h	12h	24h	48h	Coefficient of correlation (r)
Toluene	0.00±0.00	3.41±1.12^{a}	0.00±0.00	2.00±1.26^{ab}	0.00±0.00	0.00±0.00	2.00±1.64^{ab}	0.135
Hexanal	0.00±0.00	8.55±1.81	2.00±1.55	2.00±1.71	2.00±1.24	3.82±1.46	0.00±0.00	0.106
2-Ethyl-1-hexanol	2.00±1.00^{a}	2.77±0.60^{a}	2.00±1.05^{a}	2.00±1.16^{a}	2.45±1.34^{a}	0.00±0.00	0.00±0.00	−0.586**
Nonanal	0.00±0.00	3.76±1.52	4.79±1.30	2.00±1.31	2.00±1.02	2.89±1.69	0.00±0.00	0.136
2,6,10,15-Tetramethylheptadecane	0.00±0.00	9.77±1.52^{a}	2.00±1.04^{b}	2.00±1.85^{b}	2.00±1.01^{b}	0.00±0.00	0.00±0.00	−0.254
Tetradecane	2.48±1.80	4.35±1.90	2.00±1.00	2.00±1.64	2.00±1.00	2.09±1.31	0.00±0.00	−0.342
2,6,10-Trimethyltetradecane	2.18±1.62	5.95±1.25	2.00±1.01	2.00±1.93	2.00±1.04	2.36±1.33	0.00±0.00	−0.370
Benzyl hydrazinecarboxylate	0.00±0.00	0.00±0.00	2.00±1.00^{a}	0.00±0.00	0.00±0.00	0.00±0.00	0.00±0.00	−0.203
Cyclohexanone	0.00±0.00	0.00±0.00	2.06±2.20^{a}	0.00±0.00	2.00±1.81^{a}	0.00±0.00	0.00±0.00	0.045
Heptanal	0.00±0.00	0.00±0.00	2.00±1.68^{ab}	2.00±1.46^{ab}	2.00±1.03^{ab}	4.56±1.07^{a}	0.00±0.00	0.246
1-Octen-3-ol	0.00±0.00	0.00±0.00	6.00±1.30^{a}	2.00±1.44^{ab}	2.00±1.16^{ab}	0.00±0.00	0.00±0.00	0.009
2,4,6-Trimethyldecane	0.00±0.00	0.00±0.00	2.00±1.00^{a}	2.00±1.39^{a}	0.00±0.00	0.00±0.00	0.00±0.00	−0.153
2-Tridecanyl trifluoroacetate	0.00±0.00	0.00±0.00	2.00±1.02^{a}	0.00±0.00	0.00±0.00	0.00±0.00	0.00±0.00	−0.203
Dodecane	2.00±1.32^{a}	0.00±0.00	2.00±1.05^{a}	0.00±0.00	2.00±1.03^{a}	2.00±1.60^{a}	0.00±0.00	−0.123
3-Ethyl-5-(2-ethylbutyl) octadecane	0.00±0.00	0.00±0.00	2.00±1.08^{a}	0.00±0.00	0.00±0.00	0.00±0.00	0.00±0.00	−0.203
(7Z)-7-Hexadecenal	0.00±0.00	0.00±0.00	3.53±1.21^{a}	0.00±0.00	0.00±0.00	0.00±0.00	0.00±0.00	−0.162
2,4-Bis(2-methyl-2-propanyl) phenol	0.00±0.00	0.00±0.00	2.00±1.53^{a}	2.00±1.00^{a}	2.00±1.05^{a}	0.00±0.00	2.49±1.87^{a}	0.262
Heptacosane	2.46±1.75	0.00±0.00	3.36±1.08	0.00±0.00	2.00±1.00	4.75±1.22	0.00±0.00	−0.126
(14E)-14-Octadecenal	0.00±0.00	0.00±0.00	2.00±1.75^{a}	0.00±0.00	0.00±0.00	0.00±0.00	0.00±0.00	−0.203
Butyl octyl phthalate	3.73±1.38^{b}	0.00±0.00	38.70±3.53^{a}	0.00±0.00	2.00±1.28^{b}	0.00±0.00	0.00±0.00	−0.312
(2E)-2-Heptenal	0.00±0.00	0.00±0.00	0.00±0.00	2.84±1.66^{a}	0.00±0.00	0.00±0.00	0.00±0.00	0.000
2-Methyl-1-decanol	0.00±0.00	0.00±0.00	0.00±0.00	2.04±1.14^{a}	0.00±0.00	0.00±0.00	0.00±0.00	0.000
3-Pentadecanyl trifluoroacetate	0.00±0.00	0.00±0.00	0.00±0.00	2.00±1.11^{a}	0.00±0.00	0.00±0.00	0.00±0.00	0.000
2-Bromooctadecanal	2.35±1.27^{a}	0.00±0.00	0.00±0.00	2.00±1.00^{a}	0.00±0.00	0.00±0.00	0.00±0.00	−0.331
(3E)-3-(2-Propen-1-ylidene) cyclobutene	0.00±0.00	0.00±0.00	0.00±0.00	0.00±0.00	2.39±1.30^{a}	0.00±0.00	0.00±0.00	0.162
Undecane	0.00±0.00	0.00±0.00	0.00±0.00	0.00±0.00	2.00±1.11^{a}	0.00±0.00	0.00±0.00	0.203
2,6,10-Trimethyldodecane	0.00±0.00	0.00±0.00	0.00±0.00	0.00±0.00	2.00±1.14^{a}	0.00±0.00	0.00±0.00	0.203
4-Tetradecanyl trifluoroacetate	0.00±0.00	0.00±0.00	0.00±0.00	0.00±0.00	2.00±1.08^{a}	0.00±0.00	0.00±0.00	0.203
Decanal	0.00±0.00	0.00±0.00	0.00±0.00	0.00±0.00	2.00±1.91^{a}	0.00±0.00	0.00±0.00	0.203

2,6,11-Trimethyldodecane	0.00±0.00	0.00±0.00	0.00±0.00	0.00±0.00	2.00±1.01[a]	2.00±1.68[a]	0.00±0.00	0.468*
2-Methyl-2-pentadecanethiol	0.00±0.00	0.00±0.00	0.00±0.00	0.00±0.00	2.00±1.00[a]	0.00±0.00	0.00±0.00	0.203
Hexadecane	2.80±1.84	0.00±0.00	0.00±0.00	0.00±0.00	2.00±1.00	2.95±0.11	2.28±1.00	0.361
2,6,10,14-Tetramethylhexadecane	0.00±0.00	0.00±0.00	0.00±0.00	0.00±0.00	2.00±1.00[a]	2.00±1.92[a]	0.00±0.00	0.468*
Stearaldehyde	0.00±0.00	0.00±0.00	0.00±0.00	0.00±0.00	2.00±1.12[a]	0.00±0.00	0.00±0.00	0.203
(Methyldisulfanyl) methane	0.00±0.00	0.00±0.00	0.00±0.00	0.00±0.00	0.00±0.00	3.43±1.14[a]	2.54±1.06[a]	0.602**
Dimethyltrisulfane	0.00±0.00	0.00±0.00	0.00±0.00	0.00±0.00	0.00±0.00	2.00±2.00[a]	2.12±2.38[a]	0.602**
Dimethyltetrasulfane	0.00±0.00	0.00±0.00	0.00±0.00	0.00±0.00	0.00±0.00	2.44±1.67	2.17±1.60	0.602**
1H-Indole	0.00±0.00	0.00±0.00	0.00±0.00	0.00±0.00	0.00±0.00	8.29±1.36	2.00±1.28	0.654**
2-Methyl-1-butanamine	0.00±0.00	0.00±0.00	0.00±0.00	0.00±0.00	0.00±0.00	0.00±0.00	2.12±1.41[a]	0.486*
3-Methyl-1-butanol	2.00±1.00[a]	0.00±0.00	0.00±0.00	0.00±0.00	0.00±0.00	0.00±0.00	3.70±1.36[a]	−0.071
2-Octanone	2.00±1.30[ab]	0.00±0.00	0.00±0.00	0.00±0.00	0.00±0.00	0.00±0.00	3.97±1.58[a]	−0.071

Denotes: **: at the 0.01 level (two-tailed), the correlation is significant; *: at the 0.05 level (two-tailed), the correlation is significant.

Table S4. Change of relative peak area of volatile compounds in chicken during storage (mean ± SD) and correlation analysis with storage hours. (GC-MS 10^{-6} *Staphylococcus aureus* suspension)

Metabolite	0h	4h	8h	12h	24h	48h	Coefficient of correlation (r)
Toluene	2.14±1.46[a]	2.00±1.68[a]	2.00±1.03[a]	0.00±0.00	0.00±0.00	2.33±1.25[a]	−0.285
Heptanal	2.00±1.06[a]	2.00±1.00[a]	2.00±1.09[a]	2.00±1.14[a]	0.00±0.00	0.00±0.00	−0.696**
1-Octen-3-ol	2.00±1.09[b]	2.00±1.16[b]	2.00±1.05[b]	1.11±1.39[b]	0.00±0.00	0.00±0.00	−0.746**
(E)-Oct-2-enal	2.00±1.03[a]	2.00±1.01[a]	2.00±1.16[a]	2.00±1.14[a]	0.00±0.00	0.00±0.00	−0.696**
6-Methyl-1-heptanol	2.00±1.13[a]	2.00±1.03[a]	2.00±1.11[a]	0.00±0.00	0.00±0.00	0.00±0.00	−0.800**
Nonanal	2.00±1.85[a]	2.00±1.56[a]	2.00±1.26[a]	2.00±1.18[a]	0.00±0.00	0.00±0.00	−0.690**
(2E)-2-Decenal	2.00±1.83[a]	2.00±1.39[a]	2.00±1.31[a]	2.00±1.17[a]	0.00±0.00	0.00±0.00	−0.703**
Tridecanedial	2.00±1.03[a]	2.00±1.05[a]	2.00±1.18[a]	0.00±0.00	0.00±0.00	0.00±0.00	−0.807**
(2E,4E)-Deca-2,4-dienal	2.38±1.30[a]	2.00±1.01[ab]	0.00±0.00	0.00±0.00	0.00±0.00	0.00±0.00	−0.694**
3,7,11-Trimethyl-1-dodecanol	2.00±1.10[a]	2.42±1.32[a]	2.00±1.32[a]	0.00±0.00	0.00±0.00	0.00±0.00	−0.737**
2,6,10-Trimethyltetradecane	0.94±0.84[a]	0.00±0.00	1.87±1.91[a]	2.00±1.22[a]	0.00±0.00	0.00±0.00	−0.246
Stearaldehyde	2.00±1.64[a]	0.00±0.00	0.00±0.00	2.00±1.12[a]	0.00±0.00	0.00±0.00	−0.414
Methyl N-hydroxybenzenecarboximidate	0.00±0.00	2.00±1.12[a]	2.00±1.09[a]	2.00±1.15[a]	2.79±1.62[a]	0.00±0.00	−0.016
2-Pentylfuran	2.00±1.00[a]	2.00±1.02[a]	2.00±1.15[a]	0.00±0.00	0.00±0.00	0.00±0.00	−0.800**
2,6,10,15-Tetramethylheptadecane	0.00±0.00	0.95±0.86[ab]	0.00±0.00	1.76±1.53[a]	0.00±0.00	0.00±0.00	−0.12
2-Undecenal	2.46±1.74[ab]	2.00±1.61[ab]	2.00±1.33[ab]	2.00±1.17[ab]	9.55±8.33[a]	0.00±0.00	−0.212
Hexanal	0.00±0.00	2.00±1.02[a]	1.48±1.62[ab]	2.00±1.13[a]	0.00±0.00	0.00±0.00	−0.255
(E)-Non-2-enal	0.00±0.00	0.00±0.00	2.00±1.18[a]	2.00±1.17[a]	0.00±0.00	0.00±0.00	−0.004
Tetradecane	0.00±0.00	0.00±0.00	2.00±1.39[a]	2.00±1.24[a]	0.00±0.00	0.00±0.00	−0.004
Hexadecane	0.00±0.00	0.00±0.00	2.00±1.94[a]	2.00±1.49[a]	2.00±1.05[a]	2.00±1.10[a]	0.671**
Tetratetracontane	0.00±0.00	0.00±0.00	2.35±1.27[a]	0.00±0.00	0.00±0.00	0.00±0.00	−0.103
Heptacosane	0.00±0.00	0.00±0.00	4.86±1.33[a]	2.00±1.83[ab]	2.00±1.00[ab]	0.00±0.00	0.244
1-Docosanol	0.00±0.00	0.00±0.00	0.86±1.25[a]	0.00±0.00	0.00±0.00	0.00±0.00	−0.103
3-Ethyl-5-(2-ethylbutyl) octadecane	0.00±0.00	0.00±0.00	2.00±1.49[a]	2.00±1.23[a]	0.00±0.00	0.00±0.00	−0.004
Ethyl 9-tetradecenoate	0.00±0.00	0.00±0.00	0.00±0.00	0.00±0.00	1.02±1.34[a]	0.00±0.00	0.31
Ethyl myristate	0.00±0.00	0.00±0.00	0.00±0.00	0.00±0.00	2.00±1.07[a]	0.00±0.00	0.391
Methyl *(9Z)*-9-hexadecenoate	0.00±0.00	0.00±0.00	0.00±0.00	0.00±0.00	2.42±1.32[a]	2.85±1.66[a]	0.628**

Methyl *(8E,11E)*-8,11-octadecadienoate	0.00±0.00	0.00±0.00	0.00±0.00	0.00±0.00	1.75±1.82[a]	0.00±0.00	0.31
Methyl *(9Z)*-9-octadecenoate	0.00±0.00	0.00±0.00	0.00±0.00	0.00±0.00	2.00±1.00[ab]	2.78±1.61[a]	0.702**
Ethyl *(9Z,12Z)*-9,12-octadecadienoate	0.00±0.00	0.00±0.00	0.00±0.00	0.00±0.00	2.00±1.66[a]	2.00±1.04[a]	0.803**
3-Methyl-1-butanol	0.00±0.00	0.00±0.00	0.00±0.00	0.00±0.00	0.00±0.00	2.00±1.30[a]	0.651**
Dimethyltetrasulfane	0.00±0.00	0.00±0.00	0.00±0.00	0.00±0.00	0.00±0.00	2.00±1.96[a]	0.651**
1H-Indole	0.00±0.00	0.00±0.00	0.00±0.00	0.00±0.00	2.00±1.48[a]	2.00±1.11[a]	0.803**
2,4-Bis(2-methyl-2-propanyl) phenol	0.00±0.00	0.00±0.00	0.00±0.00	0.00±0.00	0.00±0.00	2.00±1.61[a]	0.651**
γ-Dodecalactone	0.00±0.00	0.00±0.00	0.00±0.00	0.00±0.00	0.00±0.00	2.00±1.20[a]	0.651**
Ethyl 9-hexadecenoate	0.00±0.00	0.00±0.00	0.00±0.00	0.00±0.00	1.19±1.44[ab]	2.00±1.59[a]	0.750**
Ethyl palmitate	0.00±0.00	0.00±0.00	0.00±0.00	0.00±0.00	2.00±1.00[a]	2.00±1.14[a]	0.810**
Ethyl *(9Z)*-9-octadecenoate	0.00±0.00	0.00±0.00	0.00±0.00	0.00±0.00	2.00±1.00[a]	2.00±1.09[a]	0.810**

Denotes: **: at the 0.01 level (two-tailed), the correlation is significant; *: at the 0.05 level (two-tailed), the correlation is significant.

Table S5. Signal intensity of volatiles detected in GC-IMS (10^{-4} *Staphylococcus aureus* suspension)

No.	Compound	RI[a]	RT[b][s]	Dt[c]	CAS	Signal intensity[d]						
						CK	0h	4h	8h	12h	24h	48h
1	Ethyl acetate	608.4	175.529	1.3402	141-78-6	450.44	1117.82	2343.55	5345.36	3734.00	4784.04	1785.63
2	Ethyl propionate	708.2	218.779	1.4539	105-37-3	18.03	63.61	19.23	79.17	283.24	1206.93	1335.64
3	Propyl butanoate	886.8	373.009	1.2642	105-66-8	220.35	513.48	385.5	846.03	656.41	1028.52	1036.62
4	Cyclohexanone	886.5	372.722	1.4602	108-94-1	206.10	176.90	711.00	782.12	719.71	891.82	72.16
5	Methacrylaldehyde	550.9	158.553	1.2143	78-85-3	74.95	50.51	229.54	202.26	129.87	189.75	16.29
6	2-Methylbutanal	732.5	232.842	1.3991	96-17-3	94.99	12.19	851.25	731.76	450.41	336.21	22.48
7	3-Butenenitrile	622.7	180.521	1.2461	109-75-1	48.25	70.99	114.57	152.85	393.31	244.41	126.20
8	1-Pentanol	766	255.107	1.5098	71-41-0	35.52	9.41	24.55	13.31	33.78	48.02	7.26
9	3-Hydroxy-2-butanone	709.9	219.691	1.3321	513-86-0	18.73	13.27	643.51	707.04	594.12	83.93	52.50
10	Hexanal	796.7	278.911	1.5632	66-25-1	262.34	35.69	172.08	35.60	325.29	1202.15	92.09
11	1-Octen-3-ol	974.4	513.251	1.1605	3391-86-4	71.02	199.58	110.74	282.04	261.54	379.28	1259.63
12	3-(Methylsulfanyl) propanal	901.5	392.657	1.4017	3268-49-3	39.56	25.55	39.27	29.89	92.34	83.62	76.62
13	2-Pentylfuran	974.6	513.587	1.2469	3777-69-3	38.80	36.44	57.86	47.86	58.07	66.16	141.79
14	Decahydronaphthalene	1042	667.186	1.2601	91-17-8	117.66	87.64	156.67	950.08	169.94	345.57	115.05
15	1-Butanol	649.8	190.976	1.3761	71-36-3	14.88	6.67	62.84	135.17	75.90	88.78	6.16
16	3-Methyl-3-buten-1-ol	724.6	228.085	1.2449	763-32-6	27.13	31.50	51.55	78.05	77.10	99.64	147.34
17	2-Hexanol	732.7	232.909	1.2755	626-93-7	53.90	75.38	35.55	59.01	113.86	305.64	1284.88
18	3-Methyl-1-butanol D	733.3	233.301	1.4961	123-51-3	35.38	30.95	44.30	526.35	721.11	866.33	184.41
19	Heptan-2-one	887.4	373.837	1.6271	110-43-0	21.48	24.37	29.06	24.22	92.96	132.62	143.52
20	(1R,5R)-2,6,6-Trimethylbicyclo[3.1.1]hept-2-ene	901.9	393.171	1.2157	80-56-8	22.75	36.55	30.88	94.93	49.19	37.53	18.59
21	Ethyl 3-methylbutanoate	853	332.876	1.6565	108-64-5	50.58	48.55	56.87	2509.75	86.84	348.48	184.92
22	Ethyl 2-methylpropanoate	759.1	250.2	1.5641	97-62-1	19.35	22.53	18.25	696.73	77.95	36.60	66.58
23	Butyl acetate	709.8	219.632	1.2376	123-86-4	14.67	12.52	16.46	85.03	21.37	23.68	16.03

24	2-Methylpropanal	624.5	181.153	1.2876	78-84-2	13.09	13.64	27.60	48.11	62.38	33.91	25.74
25	2-Methylbutanoic acid	724.6	228.043	1.2096	116-53-0	185.95	230.52	860.55	1267.52	604.82	660.72	244.79
26	2-Octanone	1024.7	623.781	1.751	111-13-7	84.86	42.34	77.09	77.84	226.32	41.43	666.42
27	Butan-2-one	551.4	158.674	1.2541	78-93-3	31.11	34.88	56.25	113.41	632.70	499.13	31.96
28	3-Methylbutanal	650	191.075	1.4108	590-86-3	39.80	5.74	564.10	409.16	281.64	122.26	7.69
29	3-Methyl-1-butanol M	745.4	240.976	1.245	123-51-3	37.74	46.11	50.45	50.04	134.60	58.74	66.04
30	1-Hydroxyacetone	703.9	216.41	1.2365	116-09-6	16.32	21.27	124.49	211.11	110.68	22.36	65.75
31	2-Methyl-1-butanol	714.1	222.016	1.2361	137-32-6	23.64	13.01	97.37	174.07	87.13	35.94	62.34
32	2-Heptanol	852.2	332.053	1.3775	543-49-7	55.38	48.24	61.61	362.60	238.00	727.39	2163.47
33	1-Penten-3-one	694.4	211.48	1.3105	1629-58-9	17.83	24.98	18.98	25.52	22.89	143.24	10.87
34	Methyl butyrate	716	223.102	1.4325	623-42-7	9.65	6.14	7.15	5.59	5.76	51.88	12.56
35	2-Pentanone	659.9	195.23	1.3726	107-87-9	21.17	7.49	58.10	99.90	60.17	84.55	10.60
36	(2E)-2-Pentenal	693	210.792	1.3663	1576-87-0	27.72	18.93	21.17	40.41	32.27	101.21	7.19
37	2-Hexanone	695	211.784	1.1912	591-78-6	130.34	60.38	85.98	193.58	76.84	290.48	185.59
38	1-Hexanol	805.1	286.094	1.3186	111-27-3	16.26	15.14	15.52	13.61	15.72	32.07	12.78
39	Hexyl acetate	1013.9	598.164	1.8922	142-92-7	554.28	502.53	567.15	686.86	976.76	530.90	3793.95
40	Aniline	975.2	514.722	1.429	62-53-3	23.94	22.27	27.11	25.93	32.46	45.93	262.10
41	Styrene	878.6	362.638	1.4202	100-42-5	7.98	7.25	7.57	9.43	11.46	16.54	20.95
42	(2E)-2-Hexen-1-ol	784	268.639	1.517	928-95-0	5.41	6.36	6.05	5.45	7.14	13.00	17.43
43	2-Methylpropanoic acid	800.6	282.188	1.3746	79-31-2	61.96	96.02	79.05	61.26	99.20	70.49	109.82
44	Acetic acid	587.7	168.865	1.1539	64-19-7	228.46	647.78	354.49	819.48	771.62	1069.94	1277.73
45	2,3,5-Trimethylpyrazine	1005.6	579.057	1.6215	14667-55-1	49.23	44.95	60.13	52.87	49.82	49.17	111.69

Denotes: M and D denote monomer and dimer, respectively; a: Retention index calculated using n-ketones C_4–C_9 as the external standard on an FC-SE-54 column; b: Retention time in the capillary GC column; c: The drift time in the drift tube; d: Signal intensity were the average. n=3.

Table S6. Signal intensity of volatiles detected in GC-IMS (10^{-6} *Staphylococcus aureus* suspension)

No.	Compound	RI[a]	RT[b][s]	Dt[c]	CAS	Signal intensity[d]						
						CK	0h	4h	8h	12h	24h	48h
1	2-Propanol	485.4	144.159	1.0897	67-63-0	717.61	787.08	666.46	487.57	3392.38	499.53	188.74
2	Acetone	487.4	144.526	1.119	67-64-1	6784.78	5042.82	6826.90	6928.64	2608.64	467.90	597.03
3	2-Ethylpyrazine	887.7	374.266	1.1568	13925-00-3	500.98	802.40	1761.54	1868.69	1255.83	157.64	926.81
4	Ethyl acetate M	608.4	175.515	1.0981	141-78-6	597.77	643.38	662.26	544.30	296.51	55.77	43.84
5	Ethyl acetate D	611.3	176.48	1.3361	141-78-6	821.78	1074.28	2893.37	4044.06	2051.77	64.97	27.85
6	2-3-Butanedione	588.9	169.238	1.1691	431-03-8	237.24	214.90	243.62	508.39	316.54	154.08	103.70
7	2-Methylbutanal M	660	195.31	1.1588	96-17-3	284.51	265.82	254.82	223.35	146.73	91.35	329.26
8	2-Methylbutanal D	660.3	195.432	1.3971	96-17-3	804.60	1003.28	1695.52	1555.38	518.02	34.62	28.97
9	3-Butenenitrile	660	195.312	1.247	109-75-1	607.16	647.85	650.94	636.50	1263.52	332.02	356.46
10	2-Pentanone	683	205.819	1.1227	107-87-9	153.12	110.06	145.96	155.90	104.77	27.87	27.99
11	Valeraldehyde	691.9	210.229	1.4233	110-62-3	44.06	135.02	169.55	52.04	40.63	12.63	10.36
12	3-Methylbutanal	649.6	190.922	1.4081	590-86-3	629.25	827.21	1997.22	1714.37	729.97	34.37	25.13
13	1-Pentanol M	766.3	255.327	1.2519	71-41-0	291.50	530.37	664.87	521.07	303.77	93.50	33.21
14	1-Pentanol D	766.7	255.641	1.5109	71-41-0	35.13	118.00	204.04	153.02	81.03	14.35	12.60
15	Hexanal M	797	279.189	1.2553	66-25-1	875.42	1129.60	1153.68	893.28	661.94	72.19	118.35
16	Hexanal D	796.3	278.561	1.5636	66-25-1	549.49	1425.41	1372.24	661.34	657.06	48.00	45.02
17	Ethyl 2-methylpropanoate	738	236.234	1.2003	97-62-1	53.26	84.32	54.63	74.16	141.32	21.98	84.63
18	1-Hexanol	871.7	354.272	1.3267	111-27-3	134.87	281.89	309.59	331.83	216.84	33.04	31.36
19	Cyclohexanone	887.1	373.493	1.4588	108-94-1	94.60	188.41	884.20	917.76	826.34	41.04	36.47
20	Benzaldehyde M	953.8	474.83	1.1529	100-52-7	413.03	288.08	592.09	653.53	173.87	65.60	318.70
21	3-(Methylsulfanyl)propanal	901.8	393.016	1.0916	3268-49-3	225.54	205.96	439.24	276.76	186.23	43.18	67.80
22	2,3-Pentanedione	690.7	209.59	1.218	600-14-6	75.02	120.24	120.71	136.95	579.10	148.17	208.92
23	Octanal	1007.7	583.782	1.4056	124-13-0	132.87	185.56	155.68	127.92	86.97	34.29	35.36

24	3-Hydroxy-2-butanone	711.1	220.335	1.332	513-86-0	73.83	76.60	947.78	4655.54	443.56	60.29	108.76
25	2-Methylpropanoic acid	779.9	265.471	1.3619	79-31-2	41.90	56.50	104.62	104.27	30.75	25.39	13.23
26	Benzaldehyde D	953.3	473.837	1.4737	100-52-7	44.22	29.96	67.94	79.95	27.07	22.56	29.08
27	(Methyldisulfanyl)methane	752.1	245.418	1.1242	624-92-0	36.61	21.78	32.40	21.33	19.20	9.25	10.31
28	Ethyl valerate	898.5	388.589	1.2648	539-82-2	31.02	45.06	80.51	167.80	325.72	19.79	43.00
29	Heptan-2-one M	887.6	374.122	1.2631	110-43-0	238.76	482.78	551.56	489.57	1529.66	77.57	211.44
30	(2E)-2-Pentenal	785.1	269.52	1.105	1576-87-0	46.81	78.09	228.80	161.70	36.82	22.42	10.53
31	2-Methylpyrazine	780.5	265.93	1.0915	109-08-0	239.27	243.80	319.29	258.80	128.67	23.53	31.82
32	2-Ethyl-1-hexanol	1041.4	665.656	1.4114	104-76-7	99.02	132.29	183.60	165.76	98.24	59.31	71.84
33	Phenylacetaldehyde	1042.1	667.275	1.2524	122-78-1	85.24	90.36	150.90	128.38	106.48	43.91	404.42
34	Heptan-2-one D	887.3	373.672	1.629	110-43-0	27.84	42.32	69.82	40.29	253.92	27.93	21.20
35	3-Methyl-1-butanol	733.6	233.497	1.4931	123-51-3	37.91	53.74	73.34	39.99	528.24	44.48	22.86
36	Butan-2-one	572	164.226	1.0515	78-93-3	1330.22	1397.31	1313.48	1453.67	565.15	255.99	76.60
37	3-Methylbutyl acetate	879.6	363.972	1.295	123-92-2	12.14	11.99	11.08	12.30	15.56	11.62	200.70
38	Heptanal	884.6	370.184	1.3198	111-71-7	19.50	29.78	33.09	39.50	40.98	11.88	78.19
39	2-Furylmethanethiol	889.5	376.495	1.3579	98-02-2	19.62	27.33	32.02	26.20	42.73	31.85	271.27
40	Styrene	889.1	376.001	1.4095	100-42-5	14.07	14.95	27.89	26.50	32.54	25.62	202.20
41	(2E)-2-Hexenal	833.3	312.31	1.1879	6728-26-3	44.83	55.96	45.29	41.49	51.01	27.19	308.64
42	2-Ethyl-3-methylpyrazine	992.5	550.391	1.1837	15707-23-0	35.90	40.37	38.07	41.84	29.98	30.08	298.70
43	Aniline	974	512.425	1.428	62-53-3	16.23	19.38	31.98	17.53	29.19	20.88	112.99
44	2-Hexanol	777.6	263.67	1.2825	626-93-7	6.76	9.00	9.29	6.61	24.71	55.73	132.98
45	Ethyl butyrate	781.3	266.542	1.2068	105-54-4	37.15	73.33	83.76	74.73	158.26	13.14	150.96
46	Propionic acid	685.8	207.16	1.2615	79-09-4	16.01	38.19	27.00	17.86	62.58	43.63	114.56

Denotes: M and D denote monomer and dimer, respectively; a: Retention index calculated using n-ketones C_4–C_9 as the external standard on an FC-SE-54 column; b: Retention time in the capillary GC column; c: The drift time in the drift tube; d: Signal intensity were the average. n=3.
